# Jointed magnetic skyrmion lattices at a small-angle grain boundary directly visualized by advanced electron microscopy

**DOI:** 10.1038/srep35880

**Published:** 2016-10-24

**Authors:** Takao Matsumoto, Yeong-Gi So, Yuji Kohno, Hidetaka Sawada, Ryo Ishikawa, Yuichi Ikuhara, Naoya Shibata

**Affiliations:** 1 Institute of Engineering Innovation, School of Engineering, The University of Tokyo, 2-11-16 Yayoi, Bunkyo-ku, Tokyo 113-8656, Japan; 2 Department of Materials Science and Engineering, Graduate School of Engineering and Resource Science, Akita University, 1-1 Tegata Gakuen-machi, Akita, Akita 010-8502, Japan; 3 JEOL Ltd., 1-2, Musashino 3-chome Akishima, Tokyo 196-8558, Japan

## Abstract

The interactions between magnetic skyrmions and structural defects, such as edges, dislocations, and grain boundaries (GBs), which are all considered as topological defects, will be important issues when magnetic skyrmions are utilized for future memory device applications. To investigate such interactions, simultaneous visualization of magnetic skyrmions and structural defects at high spatial resolution, which is not feasible by conventional techniques, is essential. Here, taking advantages of aberration-corrected differential phase-contrast scanning transmission electron microscopy, we investigate the interaction of magnetic skyrmions with a small-angle GB in a thin film of FeGe_1−x_Si_x_. We found that the magnetic skyrmions and the small-angle GB can coexist each other, but a domain boundary (DB) was formed in the skyrmion lattice along the small-angle GB. At the core of the DB, unexpectedly deformed magnetic skrymions, which appear to be created by joining two portions of magnetic skyrmions in the adjacent lattices, were formed to effectively compensate misorientations between the two adjacent magnetic skyrmion lattices. These observations strongly suggest the flexible nature of individual magnetic skyrmions, and also the significance of defect engineering for future device applications.

Interactions between electromagnetic structures and various kinds of structural defects have been targets of numbers of researches from not only fundamental but also from industrial point of view. Such interactions often result in the formation of domain boundaries (DBs; boundaries of two separate electromagnetic domains with different configurations) near structural defects^1^,^2^,^3^,^4^. DB formations can be considered as the consequence of total energy minimization in the system, and thus the unique domain structures are often created around the defects to reduce total energy. In recent years, magnetic skyrmion is one the most fascinating electromagnetic fine structures which can be formed in various materials^5^,^6^,^7^,^8^,^9^,^10^,^11^. Sizes of skyrmion range from a few to hundreds nanometers depending on the ratio between Heisenberg and Dzyaloshinskii-Moriya exchange interactions^12^,^13^. Since magnetic skyrmion is a topologically protected spin texture characterized by a “quantized” topological charge^14^, it is so stable and expected to be utilized for future memory devices featuring ultralow energy consumption^15^. Such attempts to use magnetic skyrmions as memory carriers are called *skyrmionics*, and various feasibilities are being intensively investigated both theoretically^16^,^17^,^18^ and experimentally^19^. Very recently, a new generation of nanoscale microwave oscillators using dynamical skyrmion state is also investigated^20^,^21^. However, in such practical applications, the influences of structural defects in real materials on the stability and dynamics of magnetic skyrmions remain to be elucidated. Because the sizes of magnetic skyrmion and structural defects are in nanometer scale, visualization techniques with very high spatial resolution are essential. Conventional Lorentz transmission electron microscopy (LTEM) is quite convenient for observing real-time dynamics of magnetic skyrmions with medium spatial resolution^8^,^9^,^10^,^11^,^15^. However, details of structural defects are obscured due to defocusing, which is an inherent characteristic of this technique. Therefore, the precise observation of structural defects is hampered by the overlapping Fresnel fringes in this technique. For specimens with artificially fabricated edges, a special technique in specimen preparation is shown to be effective to reduce such Fresnel fringes^22^, but this technique is not applicable to real materials and devices. In this respect, differential phase contrast transmission electron microscopy (DPC STEM) technique^23^,^24^,^25^,^26^,^27^,^28^,^29^ is very promising because of its in-focus imaging feature. Recent advance in segmented annular all-field (SAAF) detector^26^ connected via photomultiplier tubes combined with aberration correction technology has achieved highly sensitive visualization of electrostatic/magnetic structures at very high spatial resolution^27^. Equipment of an independent annular dark field (ADF) detector enables simultaneous visualization of structural defects. Furthermore, thanks to high-speed numerical processors directly connected to the detector system, a live reconstruction of the in-plane electrostatic/magnetic field vector is now feasible^28^. Taking advantages of these unique features of the technique, we are investigating the interactions of magnetic skyrmions with various structural defects, such as edges, dislocations and GBs. In our latest publication^29^, we have reported a unique DB core structure formation in skyrmion lattice induced by an edge of a crystal grain. Significance of such DBs in skyrmion lattices^9^,^30^,^31^,^32^ is increasingly recognized as they may affect the transport property in future devices using magnetic skyrmions. Very recently, multidomain skyrmion lattice state in Cu_2_OSeO_3_ was created by applying the magnetic field in direction deviating from the cubic axes of the material^33^. However, the relationships between such magnetic DBs and structural defects are not well understood. In the present study, we report the interactions of magnetic skyrmion lattice with a small-angle GB in a thin film of FeGe_1−x_Si_x_ visualized by the advanced DPC STEM technique. We used an electron probe size of 1.8 nm to resolve both magnetic skyrmions and small-angle GBs very clearly and simultaneously. In addition, we paid special attention to characterize the crystal orientation and chemical compositions of the film as described in the next paragraph and Supplementary Materials, in order to take into account the influence of crystallography.

## Results

### Characterization of the GB

**Figure 1:**
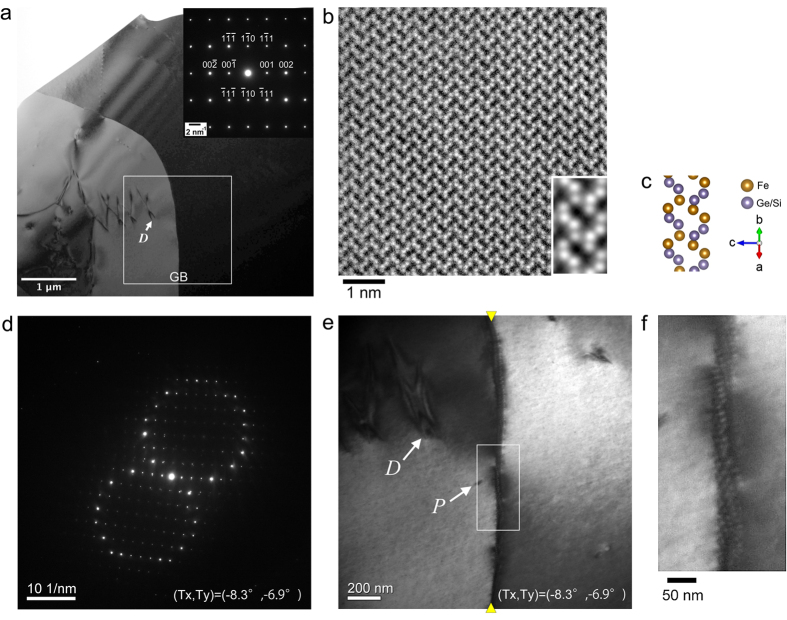
Small-angle GB in a thin film of FeGe_1−x_Si_x_ (x~0.05). (**a**) Low magnification BF TEM image of a thin film specimen containing two single crystal grains. (**b**) High-resolution [110] zone-axis HAADF STEM image of the right crystal grain. (**c**) Atomic model of Fe(Ge/Si) viewed along [110] direction. (**d**) Selected area electron diffraction pattern from the GB. Specimen titling angles are adjusted as the intermediate between zone-axis conditions of the two grains. (**e**) Corresponding BF TEM image. Edges of the boundary are designated by yellow arrowheads. The same dislocation labeled as *D* in (**a**) and a surface pit labeled as *P* are indicated by arrows. The corresponding tilting angles of double-tilt specimen holder at lower-right corners of (**d**,**e**). (**f**) An enlarged image of the boxed area in (**e**).

Figure 1a shows a low-magnification bright field (BF) TEM image of a thin film of FeGe_1−x_Si_x_ (x~0.05). There are two single crystal grains separated by an apparent curved grain boundary labeled as GB in the field-of-view. This image was recorded with the crystal orientation of the right crystal grain adjusted as [110] zone-axis as shown in the selected area electron diffraction pattern (inset). Hence, the right crystal grain appeared dark in this BF TEM image. The atomic-resolution high angle annular dark field (HAADF) STEM image (Fig. 1b) recorded from the right crystal grain with the same tilting conditions is fully consistent with B20 atomic structure of Fe(Ge,Si) viewed along [110] direction (Note that a magnified Fourier-filtered image in the bottom-right inset corresponds well with the atomic model shown in Fig. 1c). When the tilting angles of the double-tilt specimen holder were adjusted as the intermediate between zone-axis conditions of the two crystal grains, as shown in the selected-area electron diffraction pattern from the GB (Fig. 1d), the two crystal grains were imaged uniformly. Corresponding BF TEM image of the area designated by a rectangle in Fig. 1a is shown in Fig. 1e. Edges of the boundary are indicated by yellow arrowheads, and a dislocation (*D*) and a surface pit (*P*) are indicated by arrows in the figure. Note that only the dislocation is indicated in Fig. 1a. The boxed area in Fig. 1e is enlarged in Fig. 1f. As is apparent from the figure, the GB actually consists of a dense array of dislocations (additional TEM characterizations of the GB are shown in Supplementary Figs 1 and 2 and Supplementary Table 1). As for the chemical compositions of the film, we used STEM energy dispersive X-ray (EDX) analysis to confirm they are uniform across the GB (see Supplementary Figs 3 and 4). Judging from these elaborate analyses, the GB is a small-angle GB between two homogeneous B20 single crystal grains.

### Coexistence of skyrmion with GB

**Figure 2:**
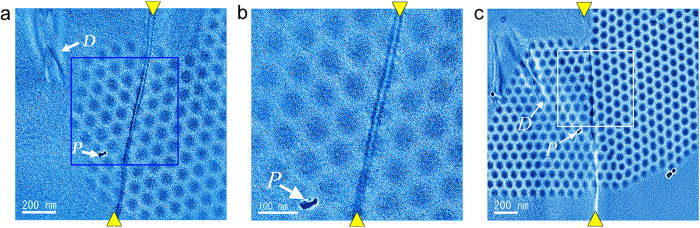
Magnetic helicity image of skyrmion lattices in the vicinity of an identical small-angle GB on two different occasions. (**a**) Skyrmion lattice is crossing the small-angle GB (designated by yellow arrowheads). A dislocation (labeled as *D*) and a surface pit (labeled by *P*) are indicated by white arrows. The region designated by a blue rectangle and the region designated by a white rectangle are magnified in (**b**). It is more evident that the lattice is crossing the small-angle GB designated by yellow arrowheads. Orientation of the skyrmion lattice is identical and continuous across the boundary. This indicates that magnetic skyrmion can coexist with the small-angle GB. (**c**) Skyrmion lattices of different orientations between the left and right structural grains are joined at the identical small-angle GB on the other occasion.

Next, we observed magnetic skyrmion lattices emerging in the vicinity of the GB by cooling the thin film from room temperature to 95 K with a nominal perpendicular magnetic field of 130 mT applied on the specimen (field-cool; FC condition). The thin film was slightly tilted (within a few degrees) to minimize diffraction contrast. The transition temperature of the film into helical magnetic phase was evaluated as 250 K from the emergence and disappearance of magnetic stripes. This value is consistent with the reported Curie temperature of the bulk^30^. Film thickness was estimated by STEM electron energy loss spectroscopy (EELS) measurement to be around 100 nm. Figure 2a shows an example of the interaction of magnetic skyrmion lattice with the GB. As reported in our previous study^29^, the magnetic helicity image was found to be quite useful for observing the interaction of magnetic skyrmion lattice with structural defects. Actually, in the magnetic helicity images as shown in Fig. 2a, hexagonal lattices of dark disk contrasts indicate clockwise rotation of the in-plane magnetization of individual magnetic skyrmion, and magnetic skyrmion lattice is imaged to cross the GB (indicated by yellow arrowheads) without changing helicity. The region designated by a blue rectangle is magnified in Fig. 2b. It is more evident that the lattice is crossing the GB (designated by yellow arrowheads), and orientation of the magnetic skyrmion lattice is identical and continuous across the boundary. Therefore, this example clearly shows that the presence of the small-angle GB has very weak effect on the structure and orientation of magnetic skyrmion lattice. In other words, magnetic skyrmion lattice can coexist with the small-angle GB.

### Jointed skyrmion at the GB

**Figure 3:**
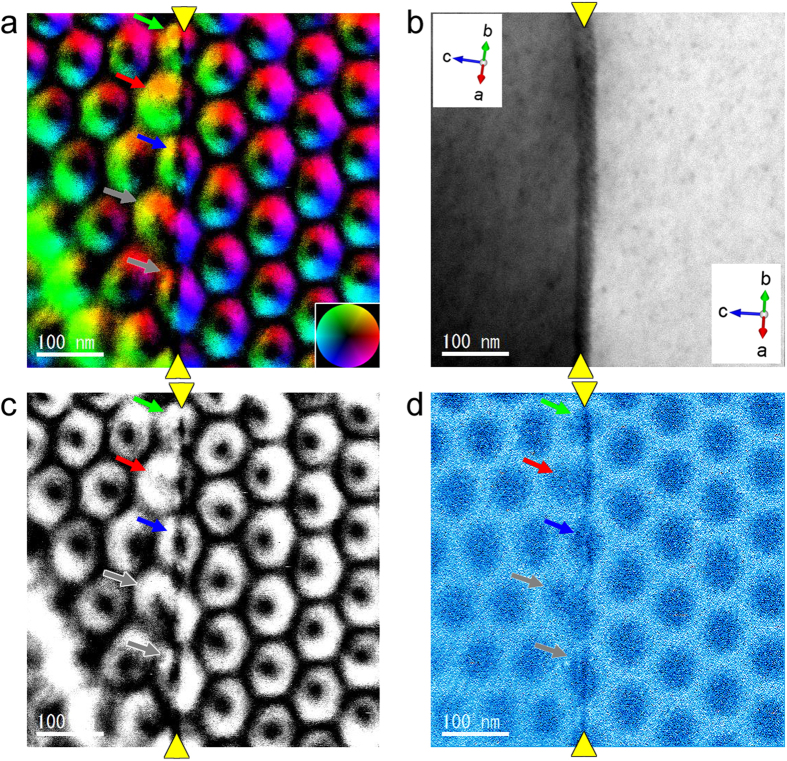
Creation of a DB of magnetic skyrmion lattices at the same small-angle GB in a thin film of FeGe1−xSix (x~0.05). (**a**) Reconstructed in-plane magnetic field vector map, (**b**) simultaneously obtained ADF image, (**c**) in-plane magnetic field intensity, and (**d**) the magnetic helicity image are shown. The small-angle GB is indicated by yellow arrowheads. Skyrmion lattices of different orientations between the left and right structural grains are observed as if partial skyrmions were jointed at the boundary. Such elongation and shrinkage of individual skyrmions were also observed in a skyrmion DB reported in our previous article, so this deformation is considered as a universal scheme of skyrmion to accommodate a constrained geometry.

After we repeated the same experiment (FC condition, 95 K, 130 mT) several times, we noticed magnetic skyrmion lattices interacted differently with the identical small-angle GB. Figure 2c shows such an example magnetic helicity image, in which a magnetic skyrmion DB was formed at the GB. The small-angle GB is indicated by the yellow arrowheads. Unlike the above example, orientations of magnetic skyrmion lattices in the two grains are different (the misorientation angle is about 30°) and strange joint of individual magnetic skyrmions at the boundary was observed in the magnetic helicity images. Note that the dislocation (*D*) and a surface pit (*P*) prove this region to be the same part of the thin film shown in Fig. 2a,b. It is evident that the magnetic skyrmion lattices of different orientations in the left and the right crystal grains appear to be jointed exactly on the small-angle GB. This strange joint is more evident in Fig. 3, which are the magnified images of the boxed area in Fig. 2c. By a careful examination, the elongated (indicated by a red arrow) or shrunk (indicated by green and blue arrows) magnetic skyrmions are formed along the small-angle GB. Such elongation and shrinkage of individual magnetic skyrmion were also observed in the magnetic skyrmion DB cores reported in our previous report^29^, which are formed inside single crystalline region of FeGe_x_Si_1−x_. In the present study, these elongated and shrunk skyrmions are positioned in the center of seven-fold and five-fold coordinated skyrmion structure units. Such tendencies of local magnetic skyrmion deformation are very similar to the DB cores formed inside single crystalline region, and thus should be universal mechanism of magnetic skyrmion lattice to accommodate a constrained geometry of DBs. However, in addition to such shape changed magnetic skyrmions, very different magnetic skyrmion structures are observed in the present case, as indicated by grey arrows in Fig. 3a,c,d. These magnetic skyrmions appear to be formed as if two partial magnetic skyrmions in the two adjacent magnetic skyrmion lattices were merged at the small-angle GB. In particular, the upper magnetic skyrmion looks like a strange joint of two partial magnetic skyrmions slightly slided vertically each other. In the DB induced by the GB, magnetic skyrmion lattices were strongly influenced by the two adjacent crystal grains up to the very vicinity of the small-angle GB, and as a result, strange skyrmions should be observed on top of the structural GB. It should be mentioned that the deformation of the magnetic skyrmion induced by the small-angle GB is not due to the variations of chemical compositions as previously reported in a literature^34^ because the chemical compositions of the film in the vicinity of the GB is uniform as evidenced by STEM EDX analysis as shown in Supplementary Figs 3 and 4. We note that such a joint and deformations are reproducible and stable during observations as demonstrated in a movie of the live DPC STEM observation (see Supplementary Movie 1 and Supplementary Fig. 5).

## Discussion

**Figure 4:**
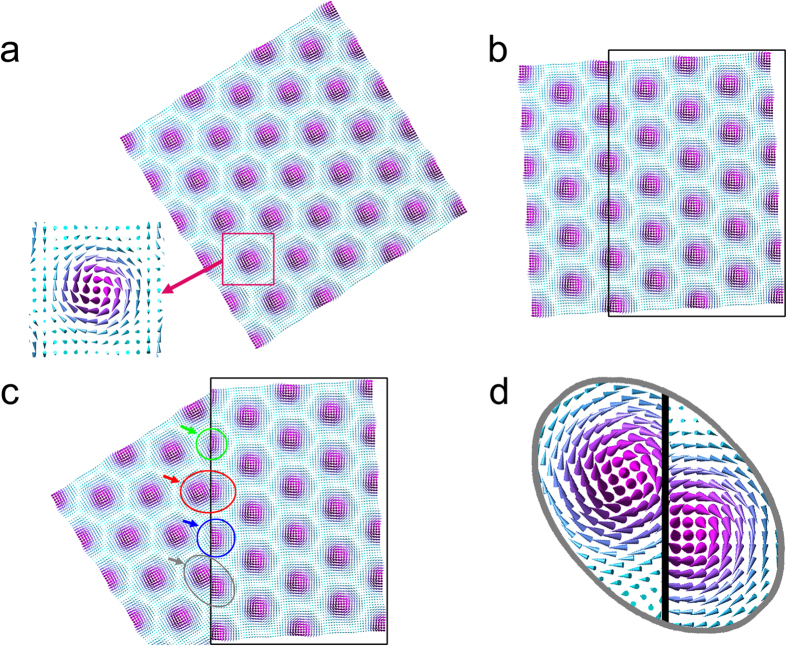
A planar schematic of the joint of two magnetic skyrmion lattices. (**a**) An original simulated magnetization of a magnetic skyrmion lattice. The magnetic field vector map is represented by three-dimensional cones as enlarged in the bottom-left inset. (**b**) A 30° clockwise rotated skyrmion lattice. (**c**) Superposition of the lattice designated by a rectangle in (**b**) over the lattice shown in (**a**). The skyrmion designated by a red circle is elongated while the ones designated by a green and a blue circle are shrunk at the boundary. In contrast, skyrmion designated by a grey circle is a strange joint as enlarged in (**d**).

**Figure 5:**
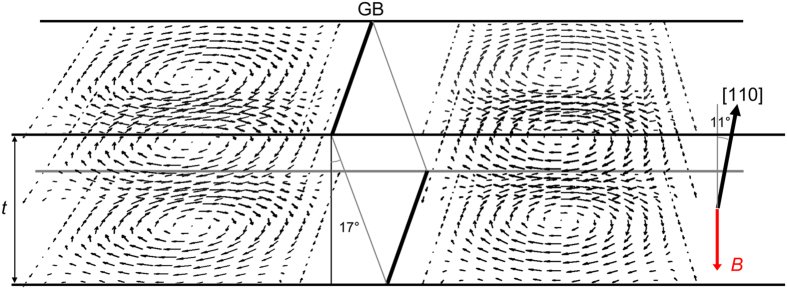
A schematic showing the three-dimensional geometry of skyrmion (reperesented by in-plane magnetic field vector arrows) and the GB. From Supplementary Table 1, the GB is tilted by 17° from the electron optical axis, while [110] zone-axis of the film is tilted by 11° in the opposite direction. A perpendicular magnetic field is applied on the thin film as designated by a red arrow as *B* in the figure.

**Figure 6:**
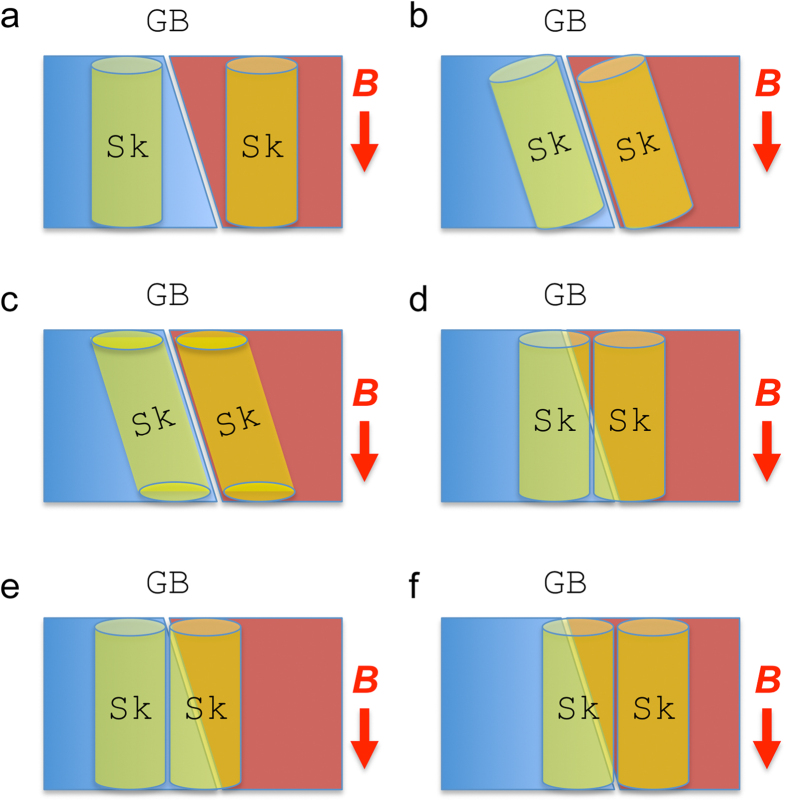
Possible cross-sectional configurations of skyrmions in the vicinity of the GB. When projected along the electron optical axis (parallel with the magnetic field B), an empty in (**a**) and overlapped regions in (**b**,**c**) are created in the vicinity of GB. Because neither empty nor overlapped region in the vicinity of GB was observed in the present experiment, these configurations appear not plausible. (**d**–**f**) Plausible configurations.

Now, we discuss the jointed skyrmion lattices using a simple planar geometric model. Figure 4 shows a schematic of the direct joint of two magnetic skyrmion lattices with 30° clockwise rotation each other. Figure 4a is an original magnetization of a magnetic skyrmion lattice. The magnetic field vector map is represented by three-dimensional cones as enlarged in the bottom-left inset. A 30° clockwise rotated magnetic skyrmion lattice is shown in Fig. 4b, and the lattice bounded by a rectangle is superimposed on the lattice of Fig. 4a to form Fig. 4c. It is evident that such a direct joint must accompany large deformations of individual magnetic skyrmion at its core. The skyrmion designated by a red circle is elongated while the ones designated by a green and a blue circles are shrunk at the boundary. Moreover, the magnetic skyrmion designated by a grey circle should be the strange joint as magnified in Fig. 4d. These simple geometric considerations are surprisingly in good agreement with the present experimental observations. This indicates that, at the structural GB, the magnetic skyrmion DB core structures should be strongly influenced by the constraint of the adjacent crystal grains. Since the magnetic skyrmions are topologically protected, they appear to keep their topology at the expense of unexpectedly large structural deformation at the DB core. Such a very flexible nature of magnetic skyrmions may be one of the reasons to stabilize DBs and defects in magnetic skyrmion lattices. It should be noted here that the three-dimensional structure of skyrmions at the boundary in the film is of interest. Based on elaborate TEM characterizations as described in Supplementary Fig. 2 and Supplementary Table 1, we analyzed the three-dimensional geometry of skyrmion and the GB as schematically shown in Fig. 5. As shown in the figure, the GB is tilted by 17° from the electron optical axis, while [110] zone-axis of the film is tilted by 11° in the opposite direction. For a straightforward interpretation of the relative configuration of the jointed skyrmion lattices and the GB, an edge-on view for both of these is preferable, which is not the case in the present work. Hence, further investigations will be necessary to define the relative configuration of jointed skyrmion lattices and a small-angle GB. We only note that several cross-sectional configurations of skyrmion and the GB in the thin film are plausible as shown in Fig. 6. When projected along the electron optical axis (parallel with the magnetic field B), an empty in Fig. 6a and an overlapped in Fig. 6b,c regions are created in the vicinity of GB. Because neither empty nor overlapped region in the vicinity of GB was observed in the present experiment, these configurations appear not plausible. Plausible configurations are shown in Fig. 6d–f.

Next, it is interesting to discuss on the unique skyrmion at the boundary in terms of the skyrmion number^14^. If such an unique skyrmion at the boundary was created by *merging* two skyrmion lattices with the same chirality, it should have the skyrmion number of two. Instead, if it was created by simply *connecting* two skyrmion lattices, it should have the skyrmion number of one. Careful examinations of the reconstructed in-plane spin texture of the strange skyrmion at the boundary as shown in Fig. 3 indicate that it has the skyrmion number of one. Therefore, it is mostly plausible that the jointed skyrmion lattices are created by a connection of two skyrmion lattices, and skyrmions at the boundary are strongly deformed as if they were created by joining two portions of skyrmions to compensate for the different orientations of the two lattices. The dynamic processes of creation of the jointed skyrmion lattices is another interesting issue to be clarified in the future.

Finally, the origin of the different skyrmion lattice structure formation at the identical small-angle GB under experimental conditions with no apparent distinction is not fully understood at present. However, we are presuming two possibilities. Firstly, the magnetic structures are known to be very sensitive to the orientation of the applied magnetic field. For example, it is well known that a magnetic bubble in some kinds of barium ferrite magnet is amenable to change its configuration in response to slight change of thin film orientation under very weak perpendicular magnetic field^35^. The recent report^33^ on the multidomain skyrmion lattice state in Cu_2_OSeO_3_ induced by applying tilted perpendicular magnetic field on the specimen exemplifies the same mechanism. Secondly, it is also well known that magnetic structures can be influenced by local strains. It has been experimentally shown that the uniaxial strain application resulted in the formation of elongated magnetic skyrmions^36^. A slight change of residual strain in a thin film may affect the creation of magnetic skyrmion lattices, particularly in the vicinity of structural defects. The interaction between local strains from the small angle GB and magnetic skyrmion lattice is not so trivial, and further experiments and theoretical simulations should be necessary to solve these issues. In any case, our observations clearly demonstrate very surprising structural flexibility and stability of individual skyrmions at lattice defect regions.

In summary, we have directly demonstrated the role of a small-angle grain boundary to create a domain boundary of magnetic skyrmion lattices, as well as the unique flexible deformation of individual skyrmions at the boundary, by using an advanced real-space visualization technique. Our study strongly indicate that the formation, orientation and local structures of skirmion lattices can be strongly influenced by the presence of a grain boundary in the materials. Our findings will stimulate further studies exploiting the significance of engineering crystal grain boundaries for realizing revolutionary skyrmionics devices in the future.

## Methods

Polycrystalline FeGe_1−x_Si_x_ was grown from FeGe_0.8_Si_0.2_ ingot by conventional solid-state reaction annealed at 600 °C for 10 days. Atomic compositions were characterized by using STEM-EDX technique. A thin film specimen was fabricated from a bulk crystal by using an Ion Slicer (IS9001, JEOL, Ltd.). Prior to observation, the thin film was further polished with a low-voltage and low-angle Ar ion beam milling apparatus (PIPS-II, Gatan, Inc.) and an ion cleaner. The thin film specimen was first characterized by using a conventional TEM (JEM-2010HC, JEOL, Ltd.). To obtain atomic resolution HAADF STEM images, we used aberration-corrected STEM (JEM-300F, JEOL, Ltd.) equipped with a cold field emission gun operated at 300 kV. For DPC STEM observations, we used a STEM (JEM-2100F, JEOL, Ltd.) equipped with a probe-forming aberration corrector (CEOS, GmbH) and a Schottky field emission gun operated at 200 kV. This microscope was equipped with a segmented annular all field (SAAF) detector which is described in a previous literature in detail. To observe magnetic skyrmion, we used a double-tilt liquid-nitrogen cooling specimen holder (Model 636, Gatan, Inc.). No temperature control of the cooling specimen holder was operated during cooling process. The thin film was slightly tilted (within a few degrees) to minimize diffraction contrast during observations. The objective lens was initially switched off, and the illumination system was adjusted to obtain a probe size of 1.8 nm with a probe-forming aperture semi-angle of 0.852 mrad. Perpendicular magnetic field was applied by slightly exciting the objective lens. The detector ranges are 0.692–1.04 mrad. and 1.56–2.70 mrad. for DPC images and ADF images, respectively.

## Additional Information

**How to cite this article**: Matsumoto, T. *et al.* Jointed magnetic skyrmion lattices at a small-angle grain boundary directly visualized by advanced electron microscopy. *Sci. Rep.* **6**, 35880; doi: 10.1038/srep35880 (2016).

## Supplementary Material

Supplementary Information

Supplementary Movie 1
