# Supplementary material for: Jointed magnetic skyrmion lattices at a small-angle grain boundary directly visualized by advanced electron microscopy
Source: Sci Rep. 2016 Oct 24;6:35880. doi: 10.1038/srep35880 (PMC5075881; doi:10.1038/srep35880)
Supplement: Supplementary Information [file srep35880-s1.pdf]

SUPPLEMENTARY INFORMATION

Jointed magnetic skyrmion lattices at a small-angle grain boundary directly visualized by advanced electron microscopy

Takao Matsumoto, Yeong-Gi So, Yuji Kohno, Hidetaka Sawada, Ryo Ishikawa, Yuichi Ikuhara & Naoya Shibata

This PDF file contains

Supplementary Fig. 1. Additional TEM Characterizations of GBs.
Supplementary Fig. 2. TEM Characterizations of the geometry of dislocation cores at the GB.
Supplementary Table 1. Resultant tilting angle and azimuth for three tilting conditions.
Supplementary Fig. 3. STEM EDX mapping of the GB.
Supplementary Fig. 4. STEM EDX line profiles of the GB.
Supplementary Fig. 5. A still image from a live movie showing the jointed magnetic skyrmion lattices at the GB.
Supplementary Movie 1. A live movie showing the jointed magnetic skyrmion lattices at the GB.

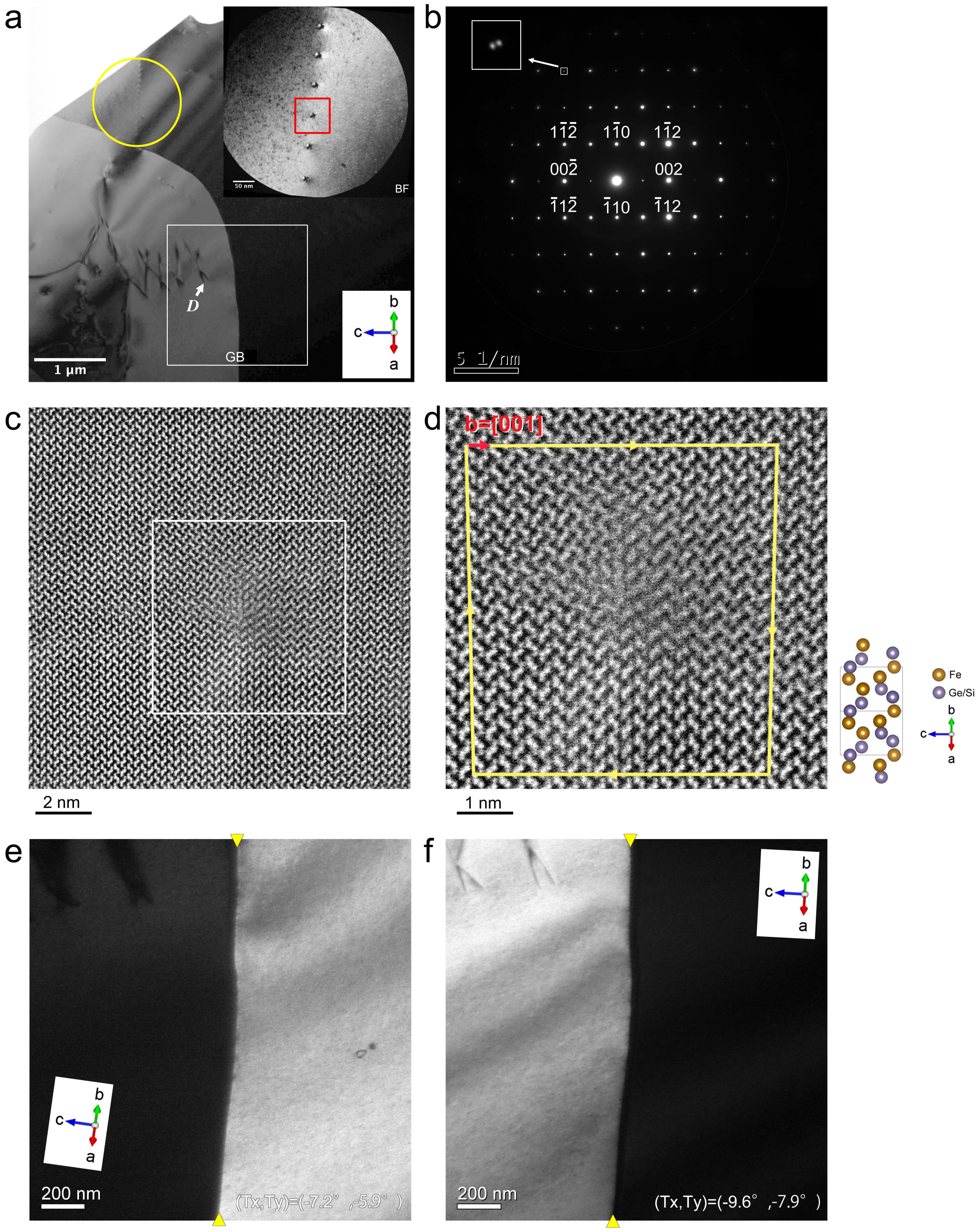

Supplementary Figure 1| Additional TEM Characterizations of GBs. (a) Low magnification BF TEM image of a (110) thin film specimen containing several GBs. There is another small-angle GB consisting of a separated array of dislocations (enlarged in the inset)

in the yellow encircled area. The small-angle GB consisting of a dense array of dislocations bounded by a white rectangle is labeled as GB. (**b**) Selected area electron diffraction pattern from the former GB. (**c**) High-resolution [110] zone-axis HAADF STEM image of the region indicated by a red rectangle in the inset in (**a**). (**d**) Higher magnification image directly shows the dislocation is an edge type with Burgers vector of b=[001] as shown by the Burgers circuit drawn in yellow. The inset shows the atomic model in [110] direction. (**e**) Corresponding BF TEM image. Directions of crystal axes of the left grain are schematically shown in the inset. (**f**) BF TEM image with the specimen tilting angles adjusted as the zone-axis condition of the right grain. Corresponding specimen tilting angles are noted at the bottom of (**e**) and (**f**), respectively. Note that the crystal axes as indicated in (**c**) and (**d**) are slightly different from the left grain.

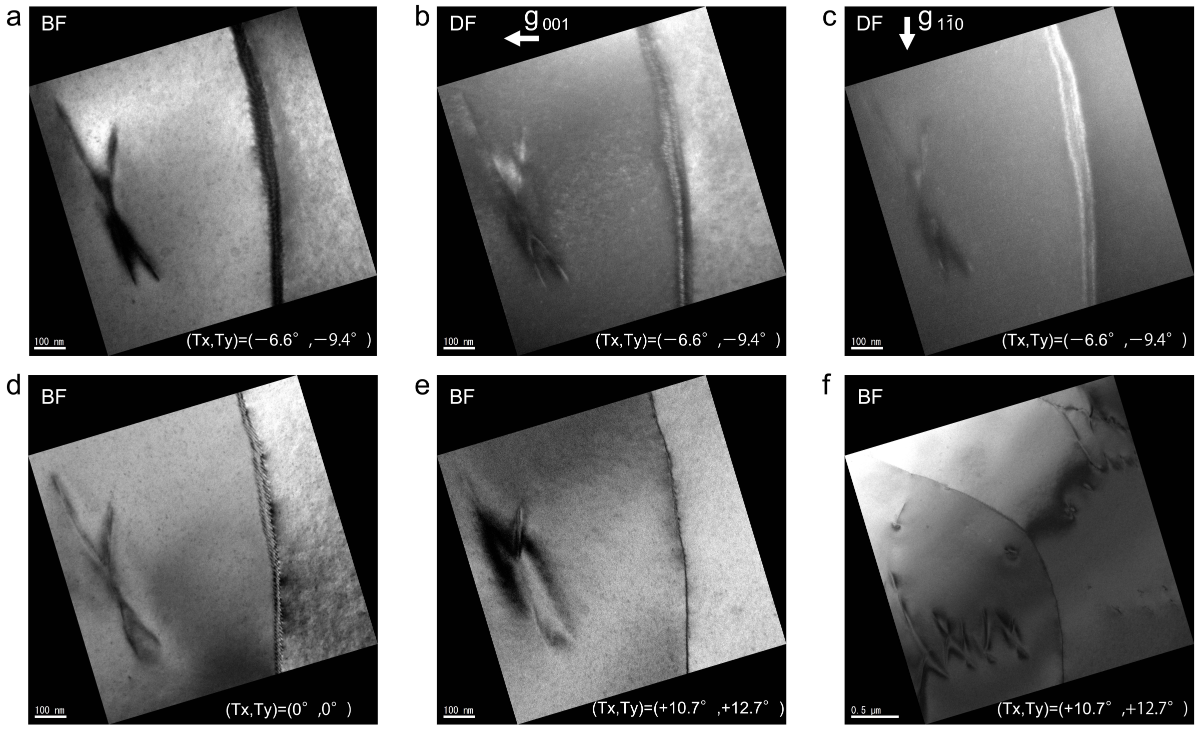

Supplementary Figure 2| TEM Characterizations of the geometry of dislocation cores at the GB. (**a**) BF TEM image of the GB in the intermediate tilting condition for the two grains separated by the GB, (**b**) DF TEM image of the GB selecting the diffraction spot corresponding to 110 reflection, (**c**) DF TEM image of the GB selecting the diffraction spot corresponding to 001 reflection. Note that contrast of dislocations in (**c**) is substantially reduced compared with their contrast in (**b**). These results are consistent to assume a Burgers vector of b=[001] for dislocations comprising the GB. (**d**) BF TEM image of the GB in the untilted condition used for DPC observations, (**e**) BF TEM image of the GB in a tilting condition to observe the GB edge-on, and (**f**) a larger field-of-view image of (**e**).

Supplementary Table 1: Tilting angles of the double-tilt specimen holder, resultant tilting angle and azimuth for three tilting conditions.

Condition/Angles	Tx	Ty	Resultant tilting angle	azimuth
Edge-on	+10.7°	+12.7°	17°	140°
Untilted (DPC)	0°	0°	0°	0°
[110] zone-axes	–6.6°	–9.4°	11°	325°

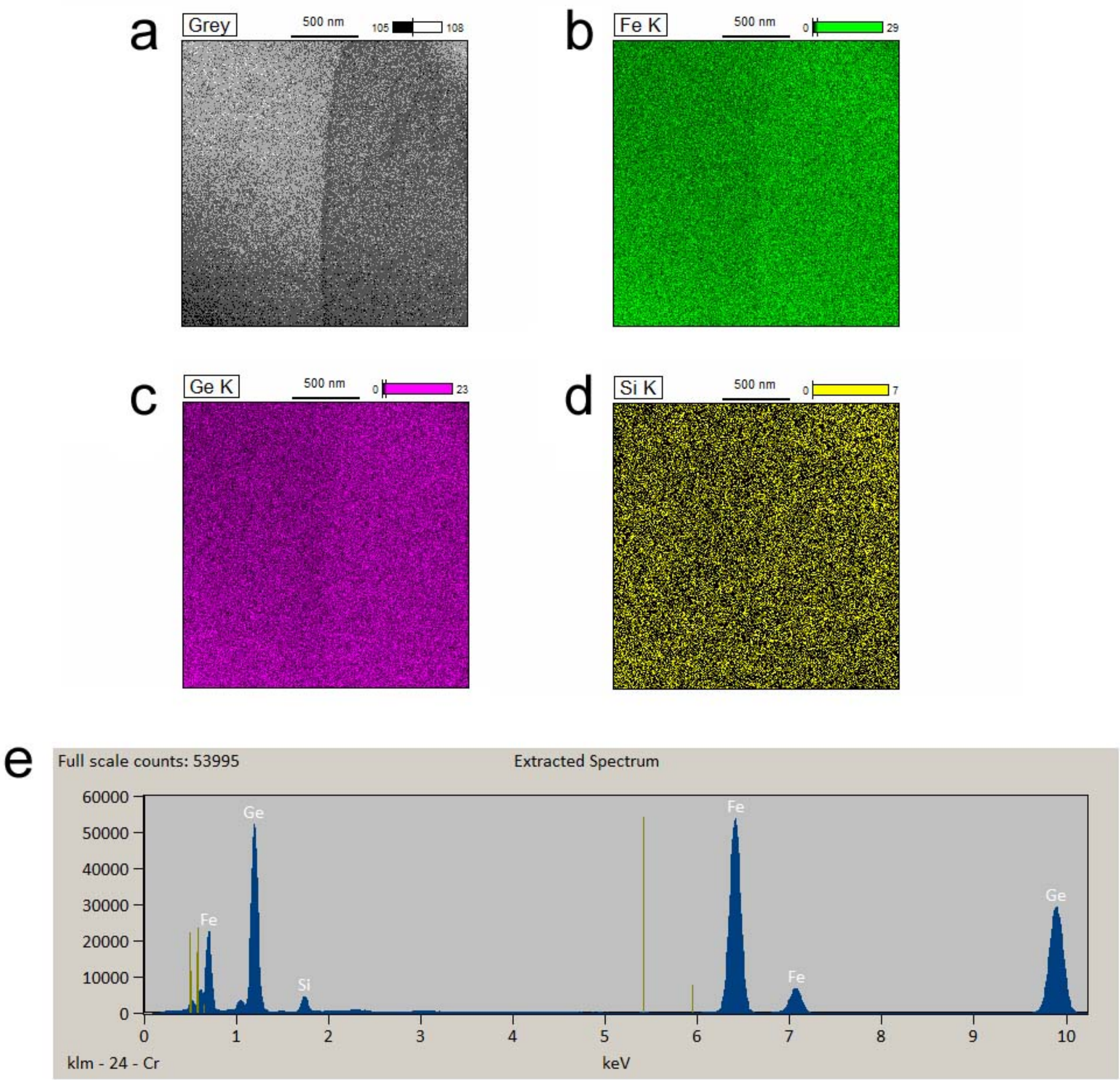

Supplementary Figure 3| STEM EDX analysis of the GB. (**a**) ADF image, (**b**) Fe, (**c**) Ge, (**d**) Si elemental map. (**e**) Extracted spectrum.

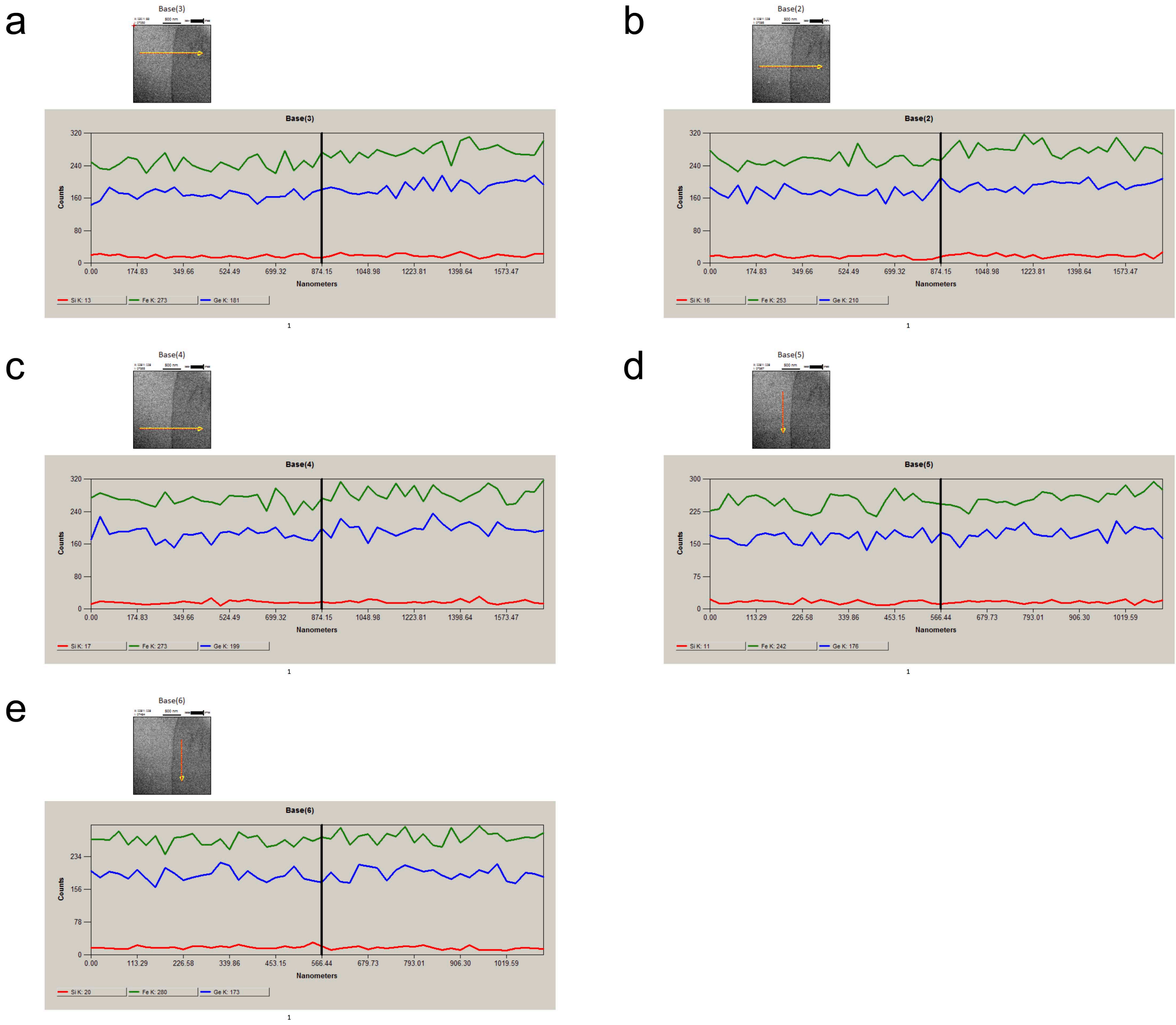

Supplementary Figure 4| STEM EDX line profiles in the vicinity of GB. (**a**)-(**e**), Several line profiles along lines as shown in each upper inset. There is no apparent difference between left and right crystal grains.

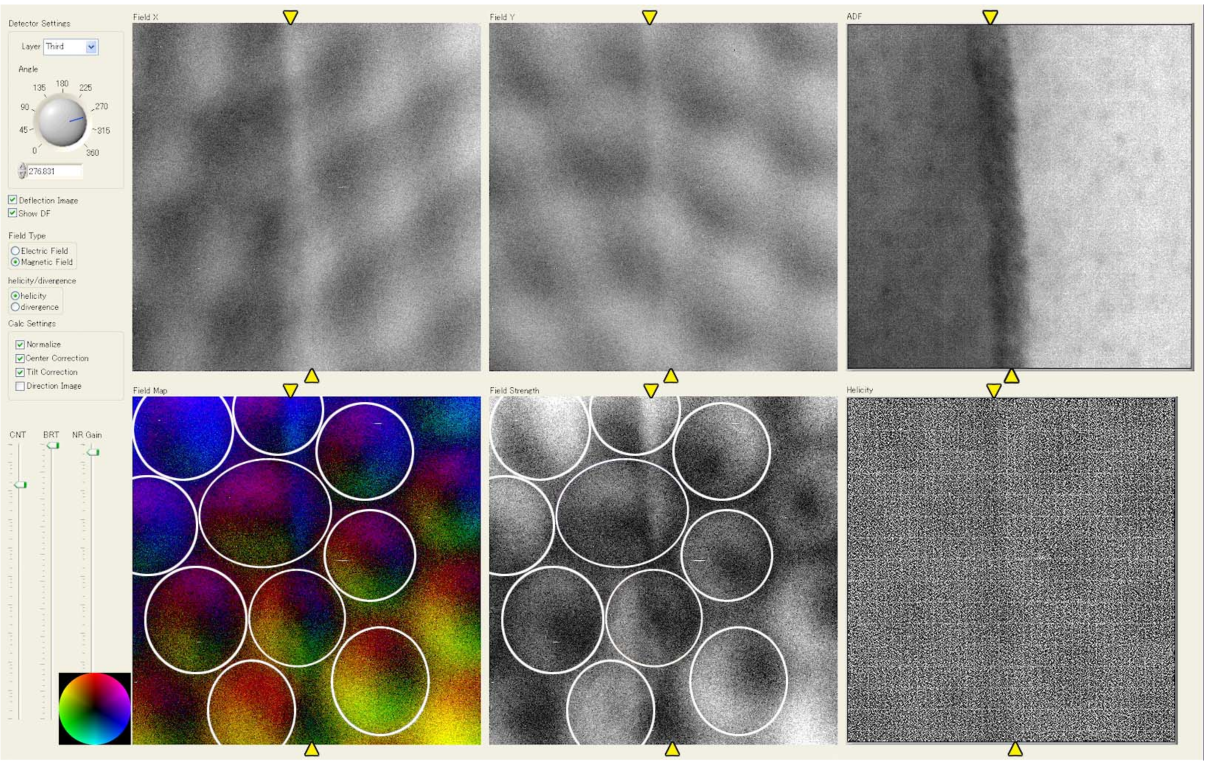

Supplementary Figure 5| A still image from a live movie showing the interation of magnetic skyrmion with the GB. Shapes of individual skyrmion are indicated by circles.
